# Evidence for an Aneugenic Mechanism of Action for Micronucleus Induction by Black Cohosh Extract

**DOI:** 10.1002/em.22334

**Published:** 2019-10-30

**Authors:** Derek T. Bernacki, Steven M. Bryce, Jeffrey C. Bemis, Stephen D. Dertinger, Kristine L. Witt, Stephanie L. Smith‐Roe

**Affiliations:** ^1^ Litron Laboratories Rochester New York; ^2^ Division of the National Toxicology Program NIEHS Research Triangle Park North Carolina

**Keywords:** dietary supplement, *Actaea racemosa*, genotoxicity, tubulin destabilizer, TK6 cells, flow cytometry

## Abstract

Black cohosh extract (BCE) is a popular botanical dietary supplement marketed to relieve symptoms of various gynecological ailments. Studies conducted by the National Toxicology Program (NTP) showed that BCE induces micronucleated erythrocytes in female rats and mice. Subsequently, the NTP showed that a variety of BCEs, including the sample that induced micronuclei (MN) *in vivo* (“NTP BCE”) had a similar effect in human TK6 cells. Further testing with the MultiFlow® DNA Damage Assay revealed that TK6 cells exposed to NTP BCE, as well as a BCE reference material (BC XRM), exhibited a signature consistent with aneugenic activity in TK6 cells. Results from experiments reported herein confirmed these *in vitro* observations with NTP BCE and BC XRM. We extended these studies to include a novel test system, the MultiFlow Aneugen Molecular Mechanism Assay. For these experiments, TK6 cells were exposed to NTP BCE and BC XRM over a range of concentrations in the presence of fluorescent Taxol (488 Taxol). After 4 h, nuclei from lysed cells were stained with a nucleic acid dye and labeled with fluorescent antibodies against phospho‐histone H3 (p‐H3) and Ki‐67. Whereas BCEs did not affect p‐H3:Ki‐67 ratios (a signature of aneugenic mitotic kinase inhibitors), 488 Taxol‐associated fluorescence (a tubulin binder‐sensitive endpoint) was affected. More specifically, 488 Taxol‐associated fluorescence was reduced over the same concentration range that was previously observed to induce MN. These results provide direct evidence that BCEs destabilize microtubules *in vitro*, and this is the molecular mechanism responsible for the aneugenicity findings. Environ. Mol. Mutagen. 2019. © 2019 The Authors. *Environmental and Molecular Mutagenesis* published by Wiley Periodicals, Inc. on behalf of Environmental Mutagen Society.

## INTRODUCTION

Due to public concern with regard to the safety of botanical dietary supplements, the National Toxicology Program (NTP) is evaluating a number of botanicals for toxicological effects (Abdel‐Rahman et al. 2011; National Toxicology Program 2017; Rider et al. 2018). One supplement that is the subject of this intense study is black cohosh extract (BCE). BCE is prepared from the root of the plant (*Actaea racemosa*) and is one of the top 10 selling botanical dietary supplements in the United States (Smith et al. 2018). It is primarily marketed to older women with the stated intention of alleviating symptoms of menopause and is represented as an alternative to hormone replacement therapy. BCE is also used by younger women with the goal of alleviating symptoms of premenstrual syndrome (PMS) and to regulate the menstrual cycle (Gafner 2016).

In 90‐day repeat‐dose rodent studies conducted by the NTP, BCE administered by oral gavage significantly increased micronucleated reticulocytes and micronucleated erythrocytes in the peripheral blood of female B6C3F1/N mice and micronucleated reticulocytes in the peripheral blood of female Wistar Han rats (Mercado‐Feliciano et al. 2012). The observation that BCE is genotoxic *in vivo*, coupled with widespread use, led the NTP to investigate whether BCE is carcinogenic the 2‐year rodent cancer bioassay (currently on test; National Toxicology Program 2019).

The toxicological evaluation of botanical materials is challenging for several reasons, including the heterogeneity of botanical preparations and adulteration with other plants or pharmaceuticals (Waidyanatha et al. 2018; Ryan et al. 2019). The NTP investigated whether preparations made from cohosh roots possess genotoxic activity in general by testing the sample of BCE (“NTP BCE”) that was genotoxic *in vivo*, 10 BCE samples from different suppliers, a BC extract reference material (BC XRM), and reference material root powders from red, yellow, and Chinese cohoshes in the *In Vitro* MicroFlow® assay, which uses flow cytometry to quantify micronuclei (MN) in mammalian cell cultures (Bryce et al. 2007). The 15 samples that were tested significantly increased MN in human lymphoblastoid TK6 cells, supporting the observations from the *in vivo* studies and suggesting that one or more genotoxic components may be common among cohosh root preparations (Smith‐Roe et al. 2018). Although the NTP has not tested BCE products that are available for purchase by consumers (ie, “finished” products such as tablets or capsules), the NTP BCE has a chromatographic profile that is very similar to the BC XRM and a BCE product, Remifemin® (Mercado‐Feliciano et al. 2012; Smith‐Roe et al. 2018).

MN are formed as the result of double‐strand DNA breaks or malsegregation of chromosome(s) (ie, clastogenicity or aneugenicity, respectively). Whereas a large proportion of clastogens are DNA‐reactive, aneugenic agents are recognized as non‐DNA reactive, most often causing genotoxicity through tubulin binding and thereby disruption of mitotic spindle function, or via inhibition of mitotic kinase(s) (Lynch et al. 2019). Knowing whether a genotoxicant has a clastogenic or aneugenic mode of action (MoA) provides useful information for risk assessment and drug discovery (Elhajouji et al. 2011; International Council for Harmonization 2011).

To gain a better understanding of how BCEs induce MN, the NTP BCE and the BC XRM samples were tested in the MultiFlow® DNA Damage Assay. This assay, also conducted using TK6 cells, uses an ensemble of machine learning (ML) algorithms to evaluate changes in several biomarkers after 4 and 24 h of exposure. The biomarker responses include phosphorylation of H2AX (γH2AX), translocation of p53 to the nucleus, phosphorylation of histone H3 at serine 10 (p‐H3), and induction of polyploidy. Together with information on cytotoxicity at 24 h, the machine‐learning algorithms translate the collection of MultiFlow responses into predictions about predominant MoA—clastogenic, aneugenic, or non‐genotoxic (Bryce et al. 2016; Bryce et al. 2017; Bryce et al. 2018; Dertinger et al. 2019). The NTP BCE and BC XRM samples were characterized by the MultiFlow assay as having aneugenic activity (Smith‐Roe et al. 2018).

Rats and mice exposed to NTP BCE in 90‐day studies also developed hematological changes consistent with megaloblastic anemia (Mercado‐Feliciano et al. 2012). Megaloblastic anemia is caused almost exclusively by insufficient levels of folate and/or cobalamin (vitamin B12) (Wickramasinghe 2006), and patients suffering from this anemia show considerably increased levels of MN (Hutchison and Ferguson‐Smith 1959; Dawson and Bury 1961), suggesting that BCE‐induced MN in rats and mice could be due to disruption of the folate metabolism pathway. Therefore, the signature of aneugenicity alone for BCE was curious, as both clastogenic and aneugenic mechanisms underlie MN arising from disruption of the folate metabolism pathway. In particular, insufficient folate reduces cellular levels of thymine, which is then replaced by uracil in DNA, resulting in chromosome breakage (Everson et al. 1988; Blount et al. 1997; MacGregor et al. 1997), and low levels of folate or cobalamin are associated with chromosome malsegregation (Fenech 2012). A follow‐up study conducted at the NTP suggested that cobalamin was dysregulated in female B6C3F1/N mice administered BCE by gavage for 3 months, but dysregulation of folate could not be ruled out (Cora et al. 2017). The solely aneugenic signature of BCE in the MultiFlow assay, possibly connected to dysregulation of cobalamin, gained plausibility when comparing the effects of BCEs to that of colchicine, a well‐characterized tubulin destabilizer that causes megaloblastic anemia by interfering with cobalamin absorption (Webb et al. 1968). The mechanism by which colchicine decreases absorption of cobalamin has not been fully described, but some studies suggest that it may interfere with vesicular transport of cobalamin by destabilizing microtubules (Stopa et al. 1979; Bose et al. 2007).

Given BCEs' *in vitro* MN‐inducing effect, the observed *in vitro* aneugenic MoA signatures, and NTP BCE's *in vivo* hematotoxicity and genotoxicity profiles, we hypothesized that cohosh extracts may exert aneugenic effects via the same mechanism responsible for colchicine‐induced genotoxicity, that is, tubulin destabilization. The experiments described herein therefore began with assessments of the inter‐laboratory reproducibility of *in vitro* MN induction by BCEs, and the aneugenic MoA predictions provided by the MultiFlow DNA Damage Assay. We then extended the work by evaluating NTP BCE and BC XRM in a new test system known as the MultiFlow Aneugen Molecular Mechanism (AMM) Assay (Bernacki et al. 2019). This TK6 cell‐based work examined changes in tubulin polymerization dynamics as well as alterations in Ki‐67 and p‐H3 labeling and, in this way, provided a means to elucidate the molecular target responsible for BCE‐induced aneugenicity.

## MATERIALS AND METHODS

### Chemicals, Cells, Culture Conditions

Dimethyl sulfoxide (DMSO, CAS no. 67‐68‐5), methotrexate (MTX, CAS no. 59‐05‐2), mebendazole (MEB, CAS no. 446‐86‐6), carbonyl cyanide *m*‐chlorophenyl hydrazone (CAS no. 555‐60‐2), carbendazim (CAS no. 2068‐78‐2), and methyl methanesulfonate (MMS, CAS no. 66‐27‐3) were purchased from Millipore Sigma, St. Louis, MO. AMG‐900 (CAS no. 877399‐52‐5) and epothilone A (CAS no. 152044‐53‐6) were from Selleckchem, Houston, TX. NTP BCE (lot number 3012872) was procured from PlusPharma (Vista, CA) and BC XRM (lot number ASB‐00030148‐005) was procured from ChromaDex (Irvine, CA). These samples of NTP BCE and BC XRM are the same materials that were tested by Smith‐Roe et al. (2018).

TK6 cells were purchased from ATCC® (cat. no. CRL‐8015) and grown in a humidified atmosphere at 37°C with 5% CO_2_ at or below 1 x 10^6^ cells/mL. The culture medium consisted of RPMI 1640 with 200 μg/mL sodium pyruvate (both from Sigma‐Aldrich, St. Louis, MO), 200 μM L‐glutamine, 50 units/mL penicillin and 50 μg/mL streptomycin (from Mediatech, Manassas, VA), and 10% v/v heat‐inactivated horse serum (Gibco®, a Thermo Fisher Scientific Company, Waltham, MA).

### *In Vitro* Micronucleus Assay: Cell Treatments, Flow Cytometric Analysis

NTP BCE and BC XRM were tested for their ability to induce MN in U‐bottom 96‐well plates (Corning, cat. no. 3799) containing 198 μL TK6 cell suspension (2 × 10^5^/mL). NTP BCE and BC XRM were prepared in DMSO as a series of 100× stock solutions. Exposure occurred by delivering 2 μL stock solution per well (duplicate wells), for final BCE concentrations of 0, 0.98, 1.95, 3.91, 7.81, 15.63, 31.25, 62.5, 125, 250, and 500 μg/mL. Concurrent positive controls were MEB (0.25 μM) and MTX (0.01 μM). Upon addition of test chemicals, the plates were immediately incubated in a humidified atmosphere at 37°C with 5% CO_2_ for 24 h. After the exposure period, cells were processed for MN frequencies via flow cytometric analysis using *In Vitro* MicroFlow® Kit reagents (Litron Laboratories, Rochester, NY). These methods have been reported in detail elsewhere (Avlasevich et al. 2006; Bryce et al. 2007).

Flow cytometric analyses were carried out using a BD FASCANTO™ II flow cytometer equipped with a BD High Throughput Sampler device (both from Becton, Dickinson and Company, San Diego, CA). Stock photomultiplier tube detectors and associated optical filter sets were used to detect fluorescence emissions associated with the MicroFlow kit‐provided fluorochromes: SYTOX Green® (detected in the FITC channel) and ethidium monoazide (EMA, PerCP‐Cy5.5 channel). The stop mode for these analyses was the acquisition of 5000 EMA‐negative nuclei per replicate well.

The NTP BCE and BC XRM micronucleus experiments described above were performed as two independent repeat experiments. Note that the same series of 100× stock solutions was used for both experiments.

### *In Vitro* Micronucleus Assay: Data Analysis

Micronucleus data analyses were restricted to those concentrations that resulted in ≤55% reduction to relative nuclei counts and did not exceed 4× increase in EMA‐positive events, whichever was lowest. Using these criteria, we were able to analyze the highest concentrations tested, that is, 500 μg/mL. The effect of treatment on mean MN frequency was evaluated with a one‐sided Dunnett's test, and *p* < 0.05 was used to indicate a significant increase over concurrent solvent control (JMP software, v12.0.1). Note that %MN were log10 transformed to satisfy homogeneity of variance assumptions (Levene's test, JMP software, v12.0.1), and data from the two independent experiments were pooled for these analyses.

Benchmark dose (BMD) analyses (exponential and Hill models) were performed with pooled NTP BCE and BC XRM MN data. The critical effect size was set to 1 (ie, 100% increase in %MN). “Compound” was selected as the covariate, and the resulting 95% confidence intervals (CIs) were used to represent the relative potency of the compounds. These methods were the same as those described previously (Dertinger et al. 2019), with the exception that the analyses described herein utilized RIVM PROAST Web software v65.2 (https://proastweb.rivm.nl; accessed June 27, 2019).

### MultiFlow DNA Damage Assay: Cell Treatments and Flow Cytometric Analysis

Chemicals were tested in U‐bottom 96‐well plates (Corning, cat. no. 3799) containing 198 μL TK6 cell suspension (2 × 10^5^/mL) plus 2 μL of DMSO‐solubilized test chemical per well. Twenty concentrations were tested using a square root 2 dilution scheme—that is, each concentration differed from the one above by a factor of 70.71%. Both BCEs were tested up to 1000 μg/mL. Each concentration was tested in a single well, whereas solvent was evaluated in four replicate wells. Concurrent positive controls were MMS and carbendazim. Upon addition of test chemicals, the plates were immediately incubated in a humidified atmosphere at 37°C with 5% CO_2_ for 24 h.

Components and preparation of the MultiFlow DNA Damage Kit—p53, γH2AX, Phospho‐histone H3 working solution were described in detail previously (Bryce et al. 2016; Bryce et al. 2017). At the 4 and 24 h sampling times, cells were resuspended with pipetting, then 25 μL were removed from each well and added to a new 96‐well plate (non‐tissue culture‐rated, Falcon, cat. no. 353910) containing 50 μL/well of pre‐aliquoted working MultiFlow reagent solution. Mixing was accomplished by pipetting the contents of each well several times. After incubation at room temperature for 30 min, samples were analyzed via flow cytometry. These methods have been reported in detail elsewhere (Bryce et al. 2016; Bryce et al. 2017).

Flow cytometric analysis was carried out using a Miltenyi Biotec MACSQuant® Analyzer 10 flow cytometer with integrated 96‐well MiniSampler device. Stock photomultiplier tube detectors and associated optical filter sets were used to detect fluorescence emissions associated with the fluorochromes: FITC (detected in the B1 channel, to use Miltenyi instrument parlance), PE (B2 channel), propidium iodide (B3 channel), and Alexa Fluor® 647 (R1 channel). Flow cytometry data were analyzed using FlowJo software v10.5.0 (Ashland, Oregon).

Representative bivariate graphs, gating logic, and position of regions were described in detail in earlier reports (Bernacki et al. 2016; Bryce et al. 2016; Bryce et al. 2017). Whereas γH2AX and p53 responses were based on the shift in median channel fluorescence intensity relative to same‐plate solvent controls, p‐H3 and polyploidy data were based on their frequency among other nuclei. Nuclei to counting bead ratios were calculated for each sample, and these ratios were used to determine absolute nuclei counts (those with 2*n* and greater DNA‐associated propidium iodide fluorescence). Nuclei counts were used to derive Relative Nuclei Counts (RNC), and %cytotoxicity was calculated as 100% minus %RNC at 24 h.

### MultiFlow DNA Damage Assay: Data Analysis

Data analyses were restricted to the lowest precipitating concentration, and those that did not exceed the assay's cytotoxicity limit. Specifically, the top concentration of each chemical had to exhibit ≤80% reduction to RNC at the 24 h time point, and a maximum of two concentrations within the cytotoxicity range 70%–80% were permitted (Dertinger et al. 2019). Based on these criteria, the top analyzable concentrations were 250 and 500 μg/mL for NTP BCE and BC XRM, respectively.

As described previously, genotoxic potential and genotoxic MoA (ie, clastogen vs. aneugen) predictions were made in two manners: comparing fold‐increase values against a global evaluation factor (GEF) rubric, and via an ML ensemble (Dertinger et al. 2019). An overall genotoxic call was made when *either* the GEF rubric or ML ensemble provided evidence of genotoxicity.

BMD analyses were performed with p‐H3 4 h data. The critical effect size was set to 0.5 (ie, 50% increase). Compound was selected as covariate, and the resulting 95% CIs were used to represent the relative potency of the compounds. As with the MN data, these analyses were performed with RIVM PROAST Web software v65.2.

Unsupervised clustering was performed for test articles predicted to have aneugenic activity by the MultiFlow DNA Damage Assay. As described previously, aneugens group broadly into two subclasses: tubulin binders vs. mitotic kinase inhibitors (Dertinger et al. 2019). The clustering analysis was performed with JMP software (v12.0.1) and utilized area‐under‐the‐curve (AUC) values based on the following four biomarkers: 4 h p‐H3, and 24 h p‐H3, p53, and polyploidy. The analysis options were set as follows: clustering method = hierarchical; method for calculating distances between clusters = “Ward”; data as usual = “Standardize robustly”; data visualization = “Dendrogram,” with “two‐way clustering.”

### MultiFlow Aneugen Molecular Mechanism Assay: Cell Treatments and Flow Cytometric Analysis

The test chemicals identified as exhibiting an aneugenic MoA were tested in a follow‐up assay designed to investigate tubulin binding vs. mitotic kinase inhibition activities. First, 488 Taxol and unlabeled Taxol supplied in prototype MultiFlow Aneugen Molecular Mechanism Kits (Litron Laboratories, Rochester, NY) were added to TK6 cells for final concentrations of 250 and 100 nM, respectively. The cells were then seeded into wells of a U‐bottom 96‐well plate (Corning, cat. no. 3799; 5 × 10^5^/mL; 198 μL/well). Suspected aneugens were added over a range of concentrations in a volume of 2 μL. A square root 2 dilution scheme was used, and four solvent control wells were included on each 96‐well plate. For these follow‐up experiments, the highest concentration was the same top analyzable concentration from the initial MultiFlow DNA Damage Assay, but in this case, cells were only treated for 4 h, and each concentration was tested in duplicate. The following concurrent controls were studied: carbendazim, AMG‐900, and epothilone A.

After 4 h of co‐treatment with 488 Taxol and suspected aneugens, cells were resuspended with pipetting, and 25 μL from each well were added to a new 96‐well plate (Falcon, cat. no. 353910) containing 50 μL/well of pre‐aliquoted lysis/labeling solution from a prototype MultiFlow—Aneugen Molecular Mechanism Kit. The proprietary lysis/labeling solution was used to simultaneously digest cytoplasmic membranes, stain chromatin with a fluorescent nucleic acid dye, and label several epitopes with fluorescent antibodies. Specifically, anti‐Ki‐67‐eFluor 660 was used as a mitotic cell marker, and anti‐phospho‐histone H3‐PE served as a mitotic marker that is responsive to Aurora kinase B inhibition. Kit‐supplied RNase and propidium iodide were used to label chromatin, and a known concentration of latex microspheres (Sphero™ Multi‐Fluorophore Particles, cat. no. FP‐3057‐2; Spherotech, Lake Forest, IL) provided a means to calculate nuclei density. After incubation at ambient temperature for 60 min in the dark, samples were analyzed via flow cytometry.

Flow cytometric analysis was performed using a MACSQuant® Analyzer 10 flow cytometer with integrated 96‐well MiniSampler device (Miltenyi Biotec, Bergisch Gladbach, Germany). As described previously (Bernacki et al. 2019), p‐H3‐positive and Ki‐67‐positive events were defined by propidium iodide–associated fluorescence (4*n* and greater DNA content) and their high‐fluorescing PE and eFluor 660 signals. The 488 Taxol biomarker was based on median channel fluorescence. For all graphical representation and statistical analyses, these values were first converted to mean fold‐change relative to a plate‐specific solvent control arithmetic mean. Gating logic required these events to exhibit propidium iodide–associated fluorescence corresponding to 2*n*–4*n* DNA content. Ki‐67‐positive cells were excluded from the median channel fluorescence calculation.

### MultiFlow Aneugen Molecular Mechanism Assay: Data Analysis

Unsupervised clustering of the responses from the AMM assay was performed with JMP software (v12.0.1) based on AUC data. To convert the biomarker responses to AUC, feature scaling was applied to every test article concentration to bring the values into the range 0 to 1 (Jayalakshmi and Santhakumaran 2011). Also, the ratio of p‐H3‐fold change to Ki‐67‐fold change was calculated for each concentration. Subsequently, p‐H3:Ki‐67 ratios were graphed against normalized concentrations to generate an AUC for each chemical. AUC values were also calculated for 488 Taxol‐fold change values vs. normalized concentrations. In all cases, AUC was calculated using Microsoft Excel via the trapezoidal rule as described at https://calculushowto.com/problem-solving/area-under-the-curve-excel (accessed June 27, 2019). Note that one (1) was subtracted from every biomarker's fold‐change value before AUC calculations were made in order to set the no effect (baseline) value to zero. With this offset in place, AUC values were zero or nearly so in the case of no response, positive in the case of an increase, and negative in the case of a reduction.

An ML prediction algorithm was developed based on an artificial neural network (ANN; JMP software, v12.0.1). The classification model was designed to categorize chemicals into one of four most likely AMM groups: tubulin destabilizers, tubulin stabilizers, Aurora kinase B inhibitors (generally these affect other aurora kinase family members as well), or a catch‐all “other” (indeterminate) group. As described by Bernacki et al. (2019), the model is based on two factors, 488 Taxol and p‐H3:Ki‐67 ratio, and three hidden nodes. The training set data were comprised of AUC values from each of 26 aneugens identified as such by the MultiFlow DNA Damage assay as reported by Bernacki et al. (2019).

## RESULTS

### *In Vitro* Micronucleus Analyses

**Figure 1:**
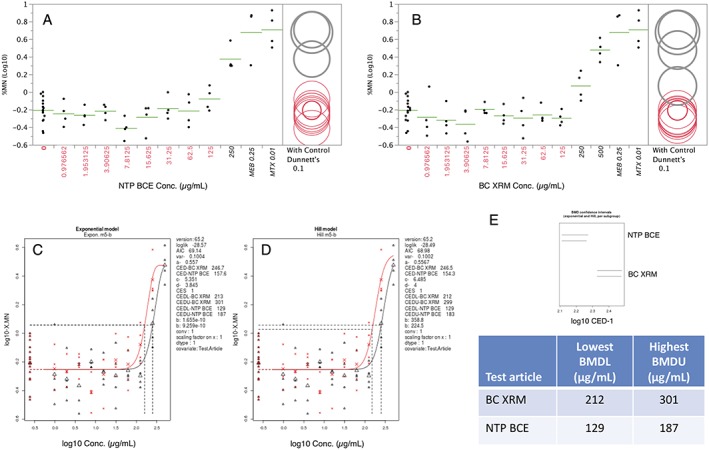
Micronucleus frequencies (log10 transformed) for TK6 cells exposed to varying concentrations of NTP BCE (a) and BC XRM (b) are shown. MEB and MTX served as positive controls and appear to the far right of each graph. Each dot represents a data point from a separate well, and the horizontal lines are mean values for each concentration. Dunnett's test results are shown to the far right of each graph, where statistically significant differences relative to the concurrent solvent control group appear as italicized black text as opposed to red text, and by a gray circle as opposed to a red circle. Circles' diameters represent 90% CIs. Panels (c–e) are output from RIVM PROAST Web software v65.2, with the lowest benchmark dose lower confidence limit (BMDL) estimate and the highest benchmark dose upper confidence limit (BMDU) value provided in the table.

TK6 cells continuously exposed to a range of NTP BCE and BC XRM concentrations were observed to exhibit significant induction of MN at the 24 h time point (Fig. 1a,b). Based on pairwise testing, the lowest effective concentration was 250 μg/mL in each case. When both exponential and Hill models are considered, BMD estimates for NTP BCE were observed to range from 154–158 μg/mL, whereas both models produced a value of 247 μg/mL for BC XRM (Fig. 1c, d). As neither exponential nor Hill model CIs overlapped (Fig. 1e), the BMD results suggest NTP BCE is slightly more potent than BC XRM.

### MultiFlow DNA Damage Assay

**Figure 2:**
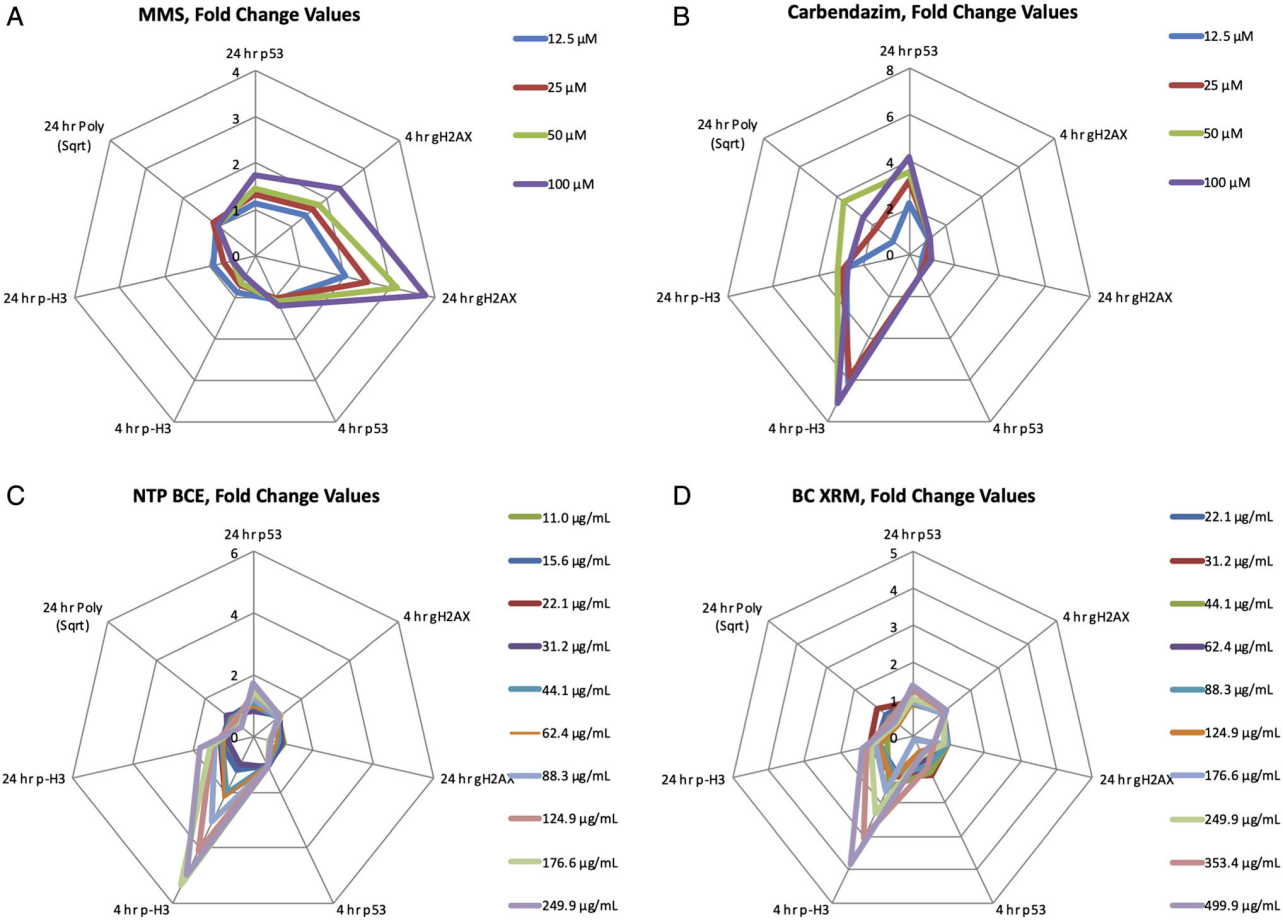
Radar plots show MultiFlow DNA Damage Assay data for seven biomarker/time point combinations and for each of four chemicals: MMS, carbendazim, NTP BCE, and BC XRM. The biomarker data are expressed as fold‐increase over mean solvent control on the same plate, and each chemical concentration appears as a different colored line. The top‐most end point (24 h p53, at 12 o'clock) is a pan‐genotoxic biomarker, whereas the biomarkers arranged on the right side of the graph are responsive to clastogens and those arranged on the left are responsive to aneugens.

TK6 cells were exposed to NTP BCE and BC XRM and evaluated for several biomarkers that are useful for distinguishing between clastogenic and aneugenic MoA. As shown by Figure 2a, b, the concurrent positive controls MMS and carbendazim exhibited prototypical clastogen and aneugen signatures, respectively. Whereas MMS was dominated by γH2AX induction and concomitant reduction in p‐H3 values, carbendazim substantially increased the frequency of p‐H3‐positive events, and at 24 h induced p53 and to some extent polyploidization. Similar to carbendazim, NTP BCE and BC XRM caused marked increases to p‐H3, especially at 4 h, and this was accompanied by p53 activation at 24 h. No evidence of γH2AX induction was observed at either time point (Fig. 2c, d).

**Figure 3:**
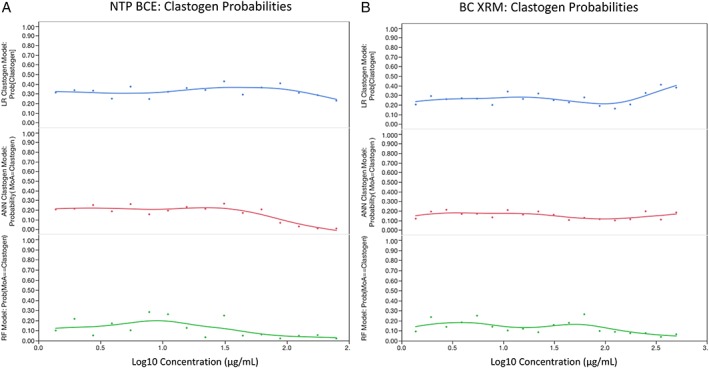
Logistic regression (LR), ANN, and random forest (RF) probabilities for clastogen classification are graphed for each NTP BCE concentration (a) and each BC XRM concentration (b). As described in Materials and Methods, two successive concentrations ≥80%, or one concentration ≥90%, is required for any one model to classify a chemical clastogenic. Two or more models must meet these requirements (simple majority) for an overall clastogen call.

**Figure 4:**
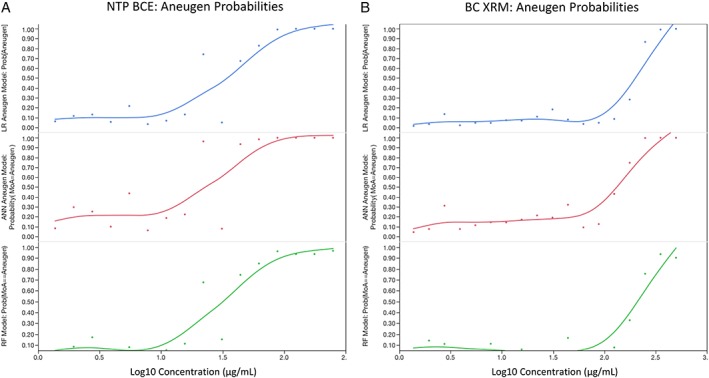
Logistic regression (LR), ANN, and random forest (RF) probabilities for aneugen classification are graphed for each NTP BCE concentration (a) and each BC XRM concentration (b). As described in Materials and Methods, two successive concentrations ≥80%, or one concentration ≥90%, is required for any one model to classify a chemical aneugenic. Two or more models must meet these requirements (simple majority) for an overall aneugen call.

NTP BCE and BC XRM data were evaluated by an ensemble of three ML prediction algorithms based on a training set of 86 chemicals (Dertinger et al. 2019). Whereas clastogenesis was not supported by any of the models (Fig. 3a,b), high probabilities of aneugenicity were found for both BCEs at several of the highest concentrations tested (Fig. 4a,b). Note that concurrent positive control chemicals exhibited expected clastogen and aneugen response profiles, and the ML ensemble returned correct MoA predictions with high probability values (>99%).

**Figure 5:**
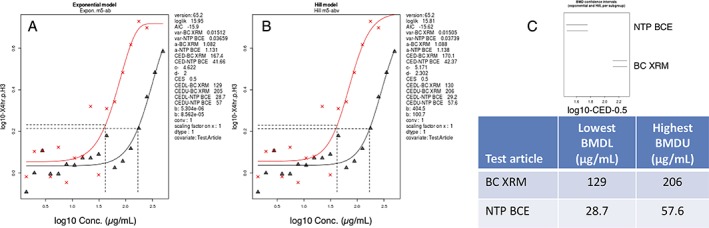
Phospho‐histone H3‐fold change values for TK6 cells exposed for 4 h to varying concentrations of NTP BCE and BC XRM are shown in (a) and (b). Panel (c) and the associated table provide output from RIVM PROAST Web software v65.2, that is the lowest benchmark dose lower confidence limit (BMDL) estimate and the highest benchmark dose upper confidence limit (BMDU) estimate.

BMD analyses for the highly responsive 4 h p‐H3 biomarker are shown in Figure 5. BMD CIs for the two BCEs did not overlap, and as with the MN end point, NTP BCE was found to be more potent than BC XRM. The p‐H3 BMD estimates (42 μg/mL and 167–170 μg/mL for NTP BCE and BC XRM, respectively) are somewhat lower than those observed for micronucleus induction, although it should be noted that different critical effect sizes were used for these end points.

**Figure 6:**
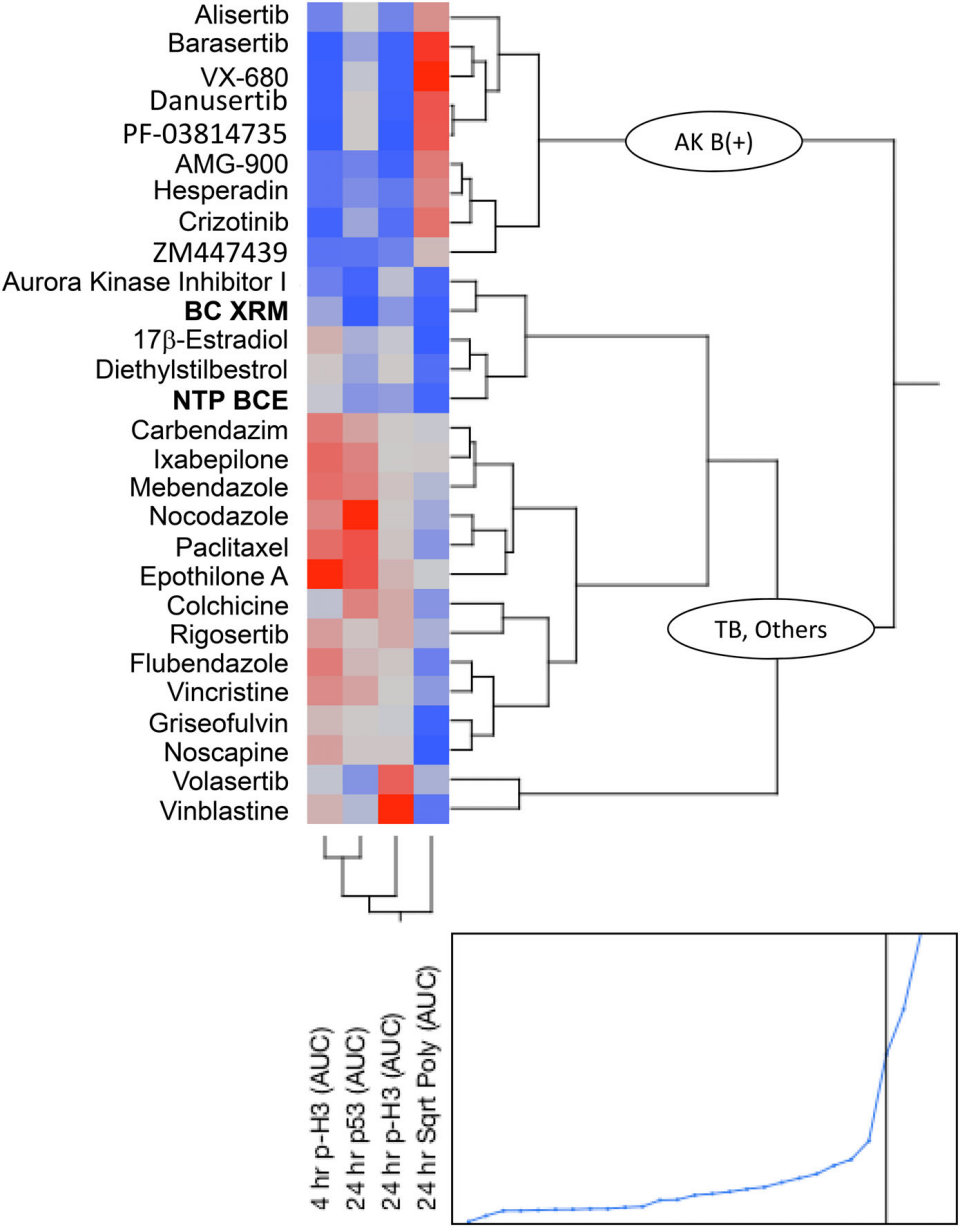
Unsupervised clustering results are shown as a two‐dimensional dendrogram for 28 chemicals that were identified as exhibiting aneugenic activity in the MultiFlow DNA Damage Assay. As described in Materials and Methods, each of the four biomarker dose–response curves was converted to an AUC for this analysis. The abbreviation “AK B(+)” denotes a clade comprised of chemicals that exhibit aurora kinase B inhibition activities, whereas “TB, Others” marks a clade primarily comprised of chemicals with tubulin binding activity, both stabilizers and destabilizers. NTP BCE and BC XRM test articles group with the “TB, Others” clade. Note the bottom‐most graph shows the horizontal distances between join points.

Dose–response data from four aneugen‐responsive biomarkers were converted to AUC values, and these were used to perform unsupervised clustering. As shown by Figure 6, the clade denoted “AK B(+)” is composed entirely of compounds known to inhibit Aurora kinase B (and typically other family members). Clade “TB, Others” is comprised of tubulin‐binders, as well as two mitotic kinase inhibitors that do not exhibit appreciable activity against aurora kinases. Both NTP BCE and BC XRM fall into the latter clade, suggestive of tubulin binding potential.

### MultiFlow Aneugen Molecular Mechanism Assay

**Figure 7:**
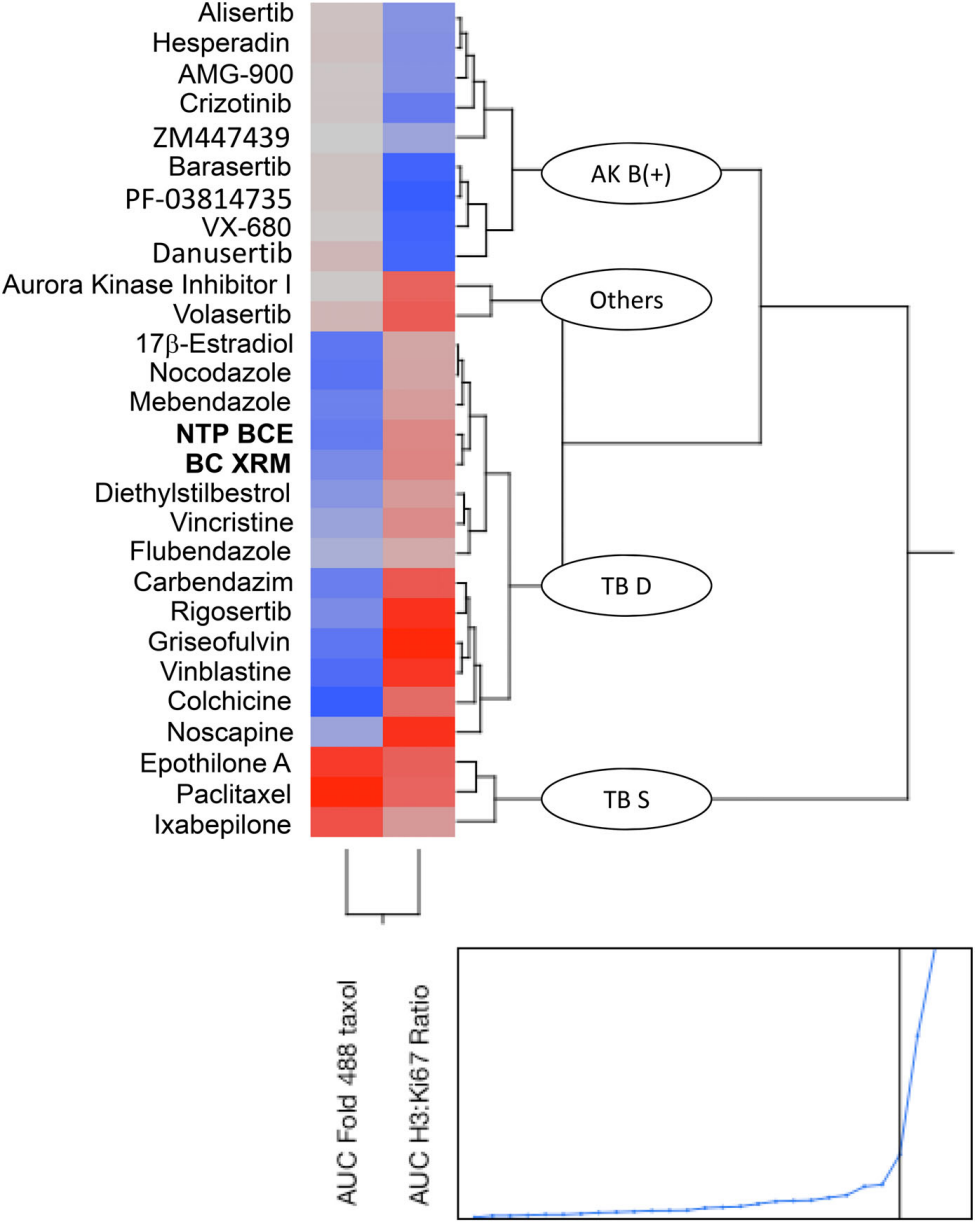
Unsupervised clustering results are shown as a two‐dimensional dendrogram for 28 chemicals. These results are based on 488 Taxol and p‐H3:Ki‐67 ratios measured in the MultiFlow Aneugen Molecular Mechanism Assay. Four clades are evident that subgroup aneugens according to molecular target: AK B(+) = Aurora kinase B inhibitors; TB S = tubulin stabilizers; TB D = tubulin destabilizers; Other = aneugenicity via other/indeterminate mechanisms. NTP BCE and BC XRM test articles group with the TD D clade. Note the bottom‐most graph shows the horizontal distances between join points.

The molecular target responsible for NTP BCE and BC XRM aneugenicity was investigated in a follow‐up assay whereby TK6 cells were exposed to a range of test article concentrations in the presence of 488 Taxol. Neither NTP BCE nor BC XRM reduced the proportion of p‐H3‐positive to Ki‐67‐positive events—i.e., the signature of aurora kinase(s) inhibition. On the other hand, they did lead to concentration‐dependent reductions to 488 Taxol‐associated fluorescence. Unsupervised clustering, provided as a two‐dimensional dendrogram in Figure 7, shows that these response patterns provide further evidence of tubulin binding properties. Furthermore, the AMM assay provided a higher degree of mechanistic resolution compared to the standard MultiFlow assay, as it distinguished between tubulin destabilizers, tubulin stabilizers, and miscellaneous (non‐Aurora kinase B) mitotic inhibitors. The two BCEs grouped with colchicine and other reference tubulin destabilizers.

Finally, AUC values from the AMM assay were applied to an artificial neural net ML classification model (Bernacki et al. 2019). The resulting predictions support the unsupervised clustering results. That is, the model predicted NTP BCE and BC XRM to be tubulin destablizers, with probabilities in excess of 99%.

## DISCUSSION

In the United States, botanical dietary supplements are used by ~18% of adults and ~5% of children, according to the 2012 National Health Interview Survey (Black et al. 2015; Clarke et al. 2015). Sales for botanical dietary supplements have increased linearly since the early 2000s, and total retail sales of these supplements exceeded 8 billion dollars in 2017 (Smith et al. 2018). However, manufacturers of dietary supplements are not required to submit efficacy and safety data to the FDA for these widely used products (Dietary Supplement Health Education Act 1994). For the past two decades, the NTP has evaluated botanical dietary supplements such as BCE in order to fill data gaps on the safety of these materials (Rider et al. 2018). The NTP discovered that BCE induced MN, a biomarker of chromosomal damage, and produced hematological perturbations that were consistent with symptoms of megaloblastic anemia in humans (Mercado‐Feliciano et al. 2012). MN and development of megaloblastic anemia can both be caused by disruption of the folate metabolism pathway. Although impairment of this pathway is primarily associated with chromosome breakage (clastogenicity), MoA studies using the MultiFlow DNA Damage assay in human TK6 cells classified two BCEs, the NTP BCE and a BC XRM, as having molecular signatures consistent with aneugenic activity (Smith‐Roe et al. 2018), an unexpected observation that was replicated in this study.

To gain a better understanding of the aneugenic activity of NTP BCE and BC XRM, we tested the same samples that were tested in the MicroFlow and MultiFlow DNA Damage assays in the novel MultiFlow AMM assay. Certain molecular events associated with chromosomal malsegregation have been very well characterized, such as disruption of tubulin polymerization and depolymerization, and inhibition of aurora kinases that are crucial for the orderly progression of mitosis (Lynch et al. 2019). The biomarkers used in the AMM assay (p‐H3, Ki‐67, and binding of 488 Taxol), exactly categorized known tubulin stabilizers, tubulin destabilizers, and aurora kinase B inhibitors, and excluded non‐aurora kinase B inhibitors (e.g., Aurora A Inhibitor I and volasertib, a Plk1 inhibitor) from these categories (Bernacki et al. 2019). The BCEs tested in this study had molecular activity profiles in the AMM assay that are consistent with tubulin destabilizers, similar to colchicine and vinca alkaloids (Fig. 7). The finding that the genotoxicity of BCEs can be attributed to an aneugenic mechanism of action is also consistent with the observation that the NTP BCE and BC XRM (and four additional BCEs selected based on limited chemical analysis) were negative in bacterial mutagenicity assays, suggesting that the genotoxicity of BCEs is likely due to a non‐DNA reactive mechanism (Smith‐Roe et al. 2018). Due to the heterogeneity of botanical preparations, the possibility remains that some BCEs could potentially have clastogenic activity. Thus far, aside from Mercado‐Feliciano et al. (2012) and Smith‐Roe et al. (2018), no other publications on the genetic toxicity of BCEs are available for comparison to this work. However, a gene expression study using livers from female Sprague‐Dawley rats administered a single dose of 37.5 mg/kg BCE suggested that it may have aneugenic activity, due to the similarity observed between the BCE gene expression profile and gene expression profiles deposited in the Iconix DrugMatrix® database from the livers of male Sprague‐Dawley rats exposed to vinca alkaloids (Einbond et al. 2012).

Taken together, our data strongly suggest that MN induced by two key BCE samples, one that exhibited genotoxic activity *in vivo* and one that is a vouchered standard reference material, are caused by destabilization of tubulin. In addition, we speculate that destabilization of tubulin could also potentially account for the megaloblastic anemia condition observed in female mice and rats exposed to BCE (Mercado‐Feliciano et al. 2012). To further investigate the relationship between BCE exposure and megaloblastic anemia, the NTP conducted a follow‐up study in female B6C3F1/N mice exposed to 1000 mg/kg/day BCE for 3 months; evidence of cobalamin deficiency was observed in these mice (Cora et al. 2017). Exposure to colchicine has also been associated with development of megaloblastic anemia in humans due to cobalamin deficiency (Webb et al. 1968), although the dependence on tubulin for uptake and cellular localization of cobalamin has not been fully characterized (Stopa et al. 1979; Bose et al. 2007).

The confirmed aneugenic activity of BCE has implications for evaluating the risk of genotoxic damage from exposure to BCE because aneugenic agents are well understood to exhibit nonlinear dose–response relationships (Elhajouji et al. 2011). More specifically, as a class, these genotoxicants have been shown to exhibit thresholds, dose levels below which no adverse effect occurs, which influences the modeling approach taken to estimate acceptable exposure levels (Elhajouji et al. 2011; MacGregor et al. 2015). Compared to linear extrapolation, which is the method most often employed for DNA‐reactive compounds, thresholded mechanisms more readily support the possibility that an adequate margin of exposure may exist that precludes the risk for genotoxic effects at real‐world exposure levels. The dose of BCE that significantly induced MN in mice was 250 mg/kg/day in 90‐day studies (Mercado‐Feliciano et al. 2012). Using allometric scaling, the human equivalent dose (HED) is 20 mg/kg. What appears to be a typical recommended dose of BCE is 40 mg/day, which also appears to be the lowest recommended dose. Assuming that a woman weighs 135 lb (61.2 kg), a dose of 40 mg/day is equivalent to 0.65 mg/kg. Therefore, the HED of 20 mg/kg is 30‐fold greater than the recommended dose of BCE at an estimate of 0.65 mg/kg for human consumption. However, recommended doses for some BCE products can be as high as 600 mg/day, and using the same estimate for a woman's weight, the difference between the HED for mice and human exposure is only twofold. We also found that the potencies of a variety of BCEs were within an order of magnitude for induction of MN in TK6 cells (Smith‐Roe et al. 2018). Therefore, the possibility remains that women could be exposed to levels of BCE that may be of concern, in terms of genotoxicity. To help address this issue, the NTP plans to use a bioassay‐directed fractionation approach to identify the tubulin‐binding component of BCE, employing the assays used in this work, and thereby determine whether real‐world dosages of specific product compositions might pose a risk to consumers.

Although BCE exhibits genotoxic potential, the use of BCE should be considered in light of risk–benefit assessments. BCE is marketed to women to alleviate symptoms of gynecological ailments, suggesting that it may contain estrogenic constituents (Gafner 2016). However, BCE lacked estrogenic activity in animal studies conducted by the NTP (Mercado‐Feliciano et al. 2012), and systematic reviews of clinical studies suggest that BCE is no more effective than placebo for alleviating symptoms of menopause (Laakmann et al. 2012; Leach and Moore 2012; Franco et al. 2016). Furthermore, in a 12‐month, randomized, double‐blinded Phase II clinical trial conducted with placebo, estrogen replacement therapy, and an authenticated BCE that had been chemically and biologically standardized, BCE performed worse than placebo for reduction of vasomotor symptoms in menopausal women (Shulman et al. 2011). Thus, the risk–benefit profile for BCE products appears weighted against benefit, and therefore, any potential adverse health risks associated with BCE should be carefully considered. Currently, the NTP is collaborating with the NIEHS Clinical Research Unit to conduct a cross‐sectional study to investigate whether women who routinely take BCE products are at risk for increased chromosomal damage and dysregulation of the folate metabolism pathway.

## Author Contributions

All authors contributed to experimental design. D.T.B. and S.M.B. performed benchtop work, and S.D.D. performed statistical analyses. S.L.S. and S.D.D. generated a first draft of the manuscript, and all authors contributed to the revisions that followed.

## Conflict of Interests

D.T.B, S.M.B, J.C.B, and S.D.D are employed by Litron Laboratories. Litron holds patents for flow cytometry‐based analyses described herein. Litron currently sells the *In Vitro* MicroFlow® Kit and the MultiFlow® DNA Damage Kit—p53, γH2AX, Phospho‐Histone H3, and intends to sell the MultiFlow® Aneugen Molecular Mechanism Kit described herein.
